# A Photo Score For Aesthetic Outcome In Sagittal Synostosis: An ERN CRANIO Collaboration

**DOI:** 10.1097/SCS.0000000000009732

**Published:** 2023-09-13

**Authors:** Linda Gaillard

**Affiliations:** Dutch Craniofacial Center, Erasmus MC–Sophia Children’s Hospital, University Medical Centre Rotterdam, Department of Plastic and Reconstructive and Hand surgery, Rotterdam, the Netherlands

**Keywords:** Sagittal synostosis, scaphocephaly, aesthetic, photo score, phenotype

## Abstract

European Reference Network (ERN) CRANIO is focused on optimizing care for patients with rare or complex craniofacial anomalies, including craniosynostosis and/or rare ear, nose, and throat disorders. The main goal of ERN CRANIO is to collect uniform data on treatment outcomes for multicenter comparison. We aimed to develop a reproducible and reliable suture-specific photo score that can be used for cross-center comparison of phenotypical severity of sagittal synostosis and aesthetic outcome of treatment. We conducted a retrospective study among nonsyndromic sagittal synostosis patients aged <19 years. We included preoperative and postoperative photo sets from 6 ERN CRANIO centers. Photo sets included bird’s eye, lateral, and anterior-posterior views. The sagittal synostosis photo score was discussed in the working group, and consensus was obtained on its contents. Interrater agreement was assessed with weighted Fleiss’ Kappa and intraclass correlation coefficients.The photo score consisted of frontal bossing, elongated skull, biparietal narrowness, temporal hollowing, vertex line depression, occipital bullet, and overall phenotype. Each item was scored as normal, mild, moderate, or severe. Results from 36 scaphocephaly patients scored by 20 raters showed kappa values ranging from 0.38 [95% bootstrap CI: 0.31, 0.45] for biparietal narrowness to 0.56 [95% bootstrap CI: 0.47, 0.64] for frontal bossing. Agreement was highest for the sum score of individual items [intraclass correlation coefficients agreement 0.69 [95% CI: 0.57, 0.82]. This is the first large-scale multicenter study in which experts investigated a photo score to assess the severity of sagittal synostosis phenotypical characteristics. Agreement on phenotypical characteristics was suboptimal (fair-moderate agreement) and highest for the summed score of individual photo score items (substantial agreement), indicating that although experts interpret phenotypical characteristics differently, there is consensus on overall phenotypical severity.

European Reference Networks (ERNs) are virtual networks of health care providers across the European Union and European Economic Area, specialized in the care of rare and/or complex disease. ERNs aim to reduce health care inequalities across Europe by pooling together disease-specific expertise, knowledge, and resources from expert centers across Europe. ERN CRANIO is focused on complex craniofacial anomalies, including craniosynostosis, and/or rare ear, nose, and throat disorders.^1^ The craniosynostosis workgroup of ERN CRANIO is focused on optimizing care for patients with craniosynostosis and their parents by comparing a multitude of clinical and esthetic outcomes between expert centers.

Sagittal synostosis is the most common form of craniosynostosis and is defined by premature closure of the sagittal suture during fetal development.^2^ Skull phenotypes of patients with craniosynostosis vary strongly depending on the affected suture. Scaphocephaly, caused by sagittal synostosis, is characterized by a long, narrow head shape with frontal bossing and an occipital bullet. Currently, there is a lack of reliable and reproducible suture-specific photo scores to assess the severity of phenotypical characteristics of scaphocephaly. Aesthetic outcomes in patients with craniosynostosis are often assessed using the Whitaker photo score or a modified version of the Whitaker score, which is focused on surgical outcomes and the need for surgical intervention rather than specific phenotypical features.^3–6^ As a result, the Whitaker score is not suited for comparing subtle differences in aesthetic outcome after intervention with different surgical techniques. In addition, the Whitaker score has been shown to have low interrater reliability.^3^

Scaphocephaly is treated with cranial vault surgery early in life to prevent intracranial hypertension and improve esthetic outcomes. Surgical techniques range from minimally invasive techniques combined with helmet therapy or springs to open cranial vault correction, of which a multitude of variations exist. The preferred standard of care has changed over the last decades and differs depending on the health care provider and within centers participating in ERN CRANIO.^7^ To accurately compare the outcome of surgical techniques, a suture-specific photo score is required. A simple and reproducible photo score based on suture-specific characteristics would be a highly valuable tool to evaluate surgical outcomes. Therefore, the aim of this study was to develop a simple, reproducible, and reliable suture-specific photo score that allows for cross-expert center comparison of phenotypical severity of scaphocephaly and postoperative aesthetic outcome.

## METHODS

### Study Design and Subjects

We conducted a retrospective study among patients with nonsyndromic sagittal synostosis aged <19 years. Six ERN CRANIO centers supplied photographs (standard 2D photographs or 2D renderings of 3D photographs) for photo score assessments: Birmingham Children’s Hospital (Birmingham, United Kingdom), Charité-Universitätsmedizin Berlin (Berlin, Germany), Erasmus Medical Center-Sophia Children’s Hospital (Rotterdam, The Netherlands), Fondazione IRCCS Istituto Neurologico Carlo Besta (Milan, Italy), Hôpital Necker-enfants-malades (Paris, France), and Hospital 12 de Octubre (Madrid, Spain).

Patients with photographs available in 4 directions (anterior-posterior view, both lateral views, and bird’s eye view) were included in the study. We included both preoperative and postoperative photo sets. Photo sets were mixed randomly. A panel of plastic surgeons and neurosurgeons evaluated photographs independently and anonymously. All panel members who participated in this study are experienced neurosurgeons or plastic surgeons who specialize in craniofacial surgery and are members of the ERN CRANIO-craniosynostosis workgroup. To qualify for membership, a minimum of 20 intracranial surgeries on patients with nonsyndromic unisutural craniosynostosis should be performed per year.

### Photo Score Development

To obtain consensus on the proposed photo score, 2 meetings were organized. During the first meeting through Microsoft Teams, a photo score was proposed based on the main scaphocephaly features. During the second meeting (in person) with the ERN CRANIO–craniosynostosis working group, the proposed score was discussed further. Items in the photo score were discussed extensively, and a small set of 8 test photo sets was scored as a pilot study using Mentimeter, a web-based survey program. The results of each photoset and inconsistencies between raters were discussed during the same meeting. The discussed characteristic scaphocephaly features were included in the final photo score.

### Photo Score Assessments

To assess the use and reliability of the proposed photo score, a panel of plastic surgeons and neurosurgeons evaluated 36 new photo sets independently and anonymously. Participants were shown photo sets through Microsoft Teams software, and photo scores were scored using Mentimeter. During this scoring session, each participant received the same instructions and was shown example photos of patients with “severe” features for each item. Example photos were based on previous consensus meetings. Participants scored 2 initial practice photo sets before rating the 36 study photo sets.All items in the photo score were scored according to the same 4-point scale, which ranged from normal to severe.

### Statistical Analysis

Statistical analyses were performed using R version 4.1.1 (August 2021). First, the interrater reliability of the entire panel was assessed with a modified version of Fleiss’ kappa with linear weights, and 95% confidence intervals were obtained using the percentile bootstrap method with 10,000 iterations.^8^ Calculations were performed using the wlin.conc() function in the package ‘raters’ in R. Second, pairwise weighted kappa analyses were performed for each combination of surgeons with the kappa2() function with equal weights of the package ‘irr’ to obtain the minimum and maximum amount of interrater reliability. The sum score was calculated by adding the scores of each individual item in the photo score. The intraclass correlation coefficient (ICC) estimates for consistency and agreement for the sum scores were obtained based on single rating, 2-way random effects models. Kappa and ICC values were interpreted according to the Landis and Koch scale.^9^ Finally, we investigated the relation between each individual item with “overall phenotype severity” assessments using Spearman correlation analyses with a 95% confidence interval obtained through bootstrapping. As a secondary analysis, we calculated the interrater reliability for high-quality photo sets. Photo sets were considered high quality when they met the following criteria: no hair obstructing the view of craniofacial features, optimal angle of the photo, and optimal lighting

## RESULTS

### Photo Score Development

**FIGURE 1:**
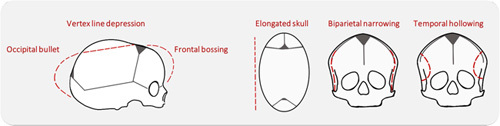
Schematic figure depicting scaphocephaly features in red dotted lines.

**FIGURE 2:**
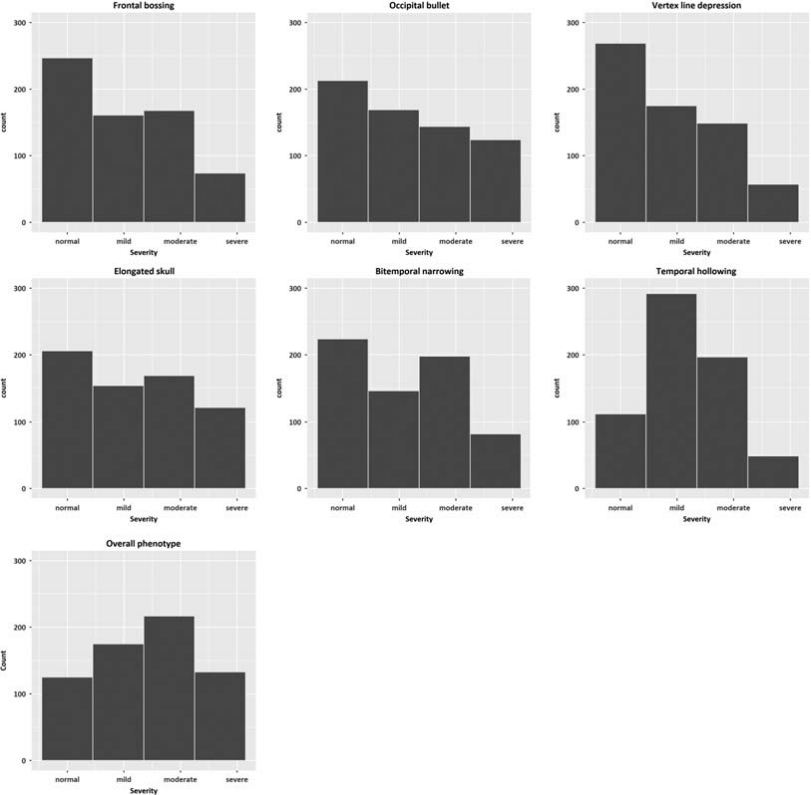
Total scores of all raters and subjects for each category.

After extensive discussion, consensus was obtained on a photo score that includes 6 characteristic scaphocephaly features (Supplemental Table 1, Supplemental Digital Content 1, http://links.lww.com/SCS/F485, Fig. 1). In addition, the overall phenotype severity was assessed separately. The severity of each item was scored on a 4-point scale with “0” indicating the absence of the described feature, “1” indicating a mild deformity, “2” indicating moderate deformity, and “3” indicating a severe deformity. For each set of photographs, the minimum score was 0 points (no abnormal features present/normal phenotype), and the maximum total score, excluding “overall phenotype,” was 18 points (all features were scored as severe). Figure 2 shows how often each item was scored as normal, mild, moderate, and severe in total.

### Photo score assessments

#### Interrater Reliability

Twenty-six surgeons participated in the photo score assessments. Twenty surgeons completed all 36 photo sets. Supplemental Table 2, Supplemental Digital Content 1, http://links.lww.com/SCS/F485 shows the interrater reliability of each item in the photo score as well the ICC of the sum score (excluding “overall phenotype”) for raters who completed scoring all photo sets. The 6 surgeons who did not complete the scoring were considered missing at random, and interrater reliability measures for the 25 photo sets that were completed by all raters were almost identical to the interrater reliability reported in Supplemental Table 2, Supplemental Digital Content 1, http://links.lww.com/SCS/F485. The weighted Fleiss’ kappa values indicate fair agreement for assessments of vertex line depression, biparietal narrowness, and temporal hollowing. There was moderate agreement for assessments of frontal bossing, occipital bullet, elongated skull appearance, and the overall phenotype. We found the best agreement for the summed score of the “frontal bossing”, “occipital bullet”, “vertex line depression”, “elongated skull appearance,” “biparietal narrowness,” and “temporal hollowing” items with the ICC indicating a substantial agreement and consistency between raters (Supplemental Table 2B, Supplemental Digital Content 1, http://links.lww.com/SCS/F485).

**FIGURE 3:**
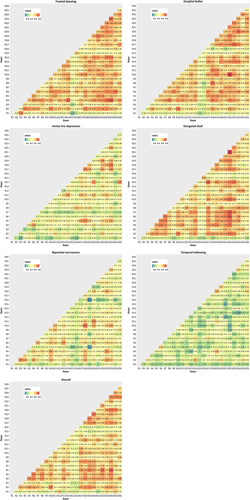
Pairwise kappa analyses. Each square represents 1 pair of raters, with the color of the square indicating the amount of agreement as shown in the legend and the number in the square indicating the κ value. Abbreviations: R indicates rater.

The pairwise weighted Kappa analyses are shown in Fig. 3. Our results indicate a considerable variation in the amount of agreement between raters. Kappa values indicate degrees of agreement that range from poor agreement to almost perfect agreement (frontal bossing (κ min=−0.05, κ max = 0.84), occipital bullet (κ min = 0.04, κ max = 0.90), vertex line depression (κ min = −0.07, κ max = 0.76), elongated skull appearance (κ min = 0.04, κ max = 0.90), biparietal narrowness (κ min = −0.17, κ max = 0.79), temporal hollowing (κ min = −0.10, κ max = 0.62), and overall phenotype (κ min = 0.07, κ max = 0.83).

#### Relation Between Individual Items and Overall Phenotype Assessment

**FIGURE 4:**
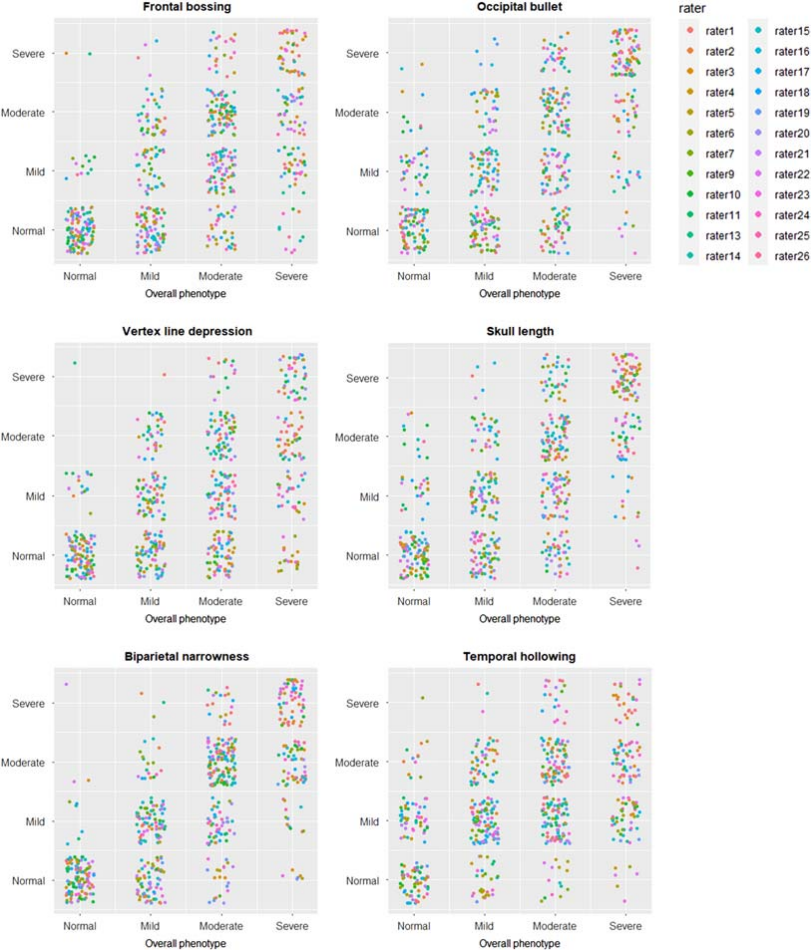
Relation of individual items with overall phenotype. Scatterplots show the scores from all raters for all subjects.

Scatter plots to indicate the relation between individual items and overall phenotype are shown in Fig. 4, and Spearman rank correlation coefficients (r_s_) for each rater are shown in Supplemental Table 3, Supplemental Digital Content 1, http://links.lww.com/SCS/F485. The correlation strength varied heavily between raters and also differed between photo score items. An elongated skull appearance (*r*_s_ min = 0.57, *r*_s_ max = 1.00) and biparietal narrowness (*r*_s_ min = 0.48, *r*_s_ max = 0.92) had the strongest positive correlation with overall phenotype, followed by the occipital bullet (*r*_s_ min = 0.32 , *r*_s_ max = 0.86) and frontal bossing (*r*_s_ min = 0.39, *r*_s_ max = 0.84). Temporal hollowing had the lowest correlation with overall phenotype (*r*_s_ min = 0.00, *r*_s_ max = 0.74), with 15 raters having a weak or very weak positive correlation between temporal hollowing and overall phenotype.

#### Subanalysis of High-Quality Photo Sets

In a secondary analysis, we investigated if agreement improved if a subset of the highest-quality photographs were selected. The kappa statistic and ICC for the highest-quality photo sets are shown in Supplemental Table 4, Supplemental Digital Content 1, http://links.lww.com/SCS/F485. Although we only investigated a small sample of photo sets, agreement on most items was similar to the total group of photo sets. However, the agreement on “frontal bossing” was substantially less in the high-quality photo sets.

## DISCUSSION

The aim of this study was to develop and investigate the performance of a simple suture-specific photo score for shape analysis in sagittal synostosis within a large group of experienced craniofacial plastic surgeons and neurosurgeons. The current study demonstrated inconsistency in scoring between experts on individual photo score items. This indicates that despite identical instructions and consensus on the photo score, experts continue to have their personal interpretation of the specific scaphocephaly characteristics.

Previous studies on aesthetic outcomes in scaphocephaly have often used a general assessment of skull/head shape or used scores that only assess aesthetic outcomes after surgical intervention and the need for re-intervention.^3–6,10–13^ However, literature on photo scores that assess phenotypical severity of specific scaphocephaly features is limited, and to our knowledge, only 2 prior studies investigated such photo scores.^14,15^ First, Van Veelen et al^15^ used a scaphocephaly photo score to assess aesthetic outcomes after spring-assisted strip craniotomy by comparing preoperative and postoperative photo score assessments. “Width”, “length”, “frontal bossing”, “occipital protuberance”, “temporal pinching”, and “vertex height” were scored on a 3-point scale. ICCs ranged from 0.4 to 0.7 for individual items as compared with a κ value of 0.4 to 0.6 for individual items in our study. Van Veelen et al found an ICC greater than 0.7 for the summed score of the individual photo score items both preoperatively and postoperatively, which is similar to the ICCs we obtained for the summed score (ICC agreement =0.7 and ICC consistency =0.8). The lower κ value of the individual photo score items in our study is likely due to the multicenter study design. In single-center studies, team members are more likely to agree on their definition of specific phenotypical characteristics and consequently have higher agreement on photo scores.

The second study on a scaphocephaly-specific photo score compared aesthetic outcomes before and after surgery by grading “ narrow elongated skull’, “frontal bossing”, “temporal pinching”, “ occipital bullet,” and “overall shape.”^14^ They did not include craniofacial surgeons in their panel, but a panel of plastic surgery trainees (N=16) and non-craniofacial consultants (N=6) scored on a scale from 0 to 100. The authors found low ICCs for both non-craniofacial consultants and registrars on all items.

A major strength of our study is its multicenter design. However, photographs were taken through center-specific standard clinical protocols, and the quality of the photo sets varied considerably. Some scaphocephaly characteristics, such as temporal hollowing and vertex line depression, are easily obscured by hair, making them difficult to assess. Nonetheless, interrater reliability did not improve when only investigating a subgroup of high-quality photo sets, implying that suboptimal interrater reliability is caused by a true difference of opinion rather than suboptimal quality photographs.

Our study illustrates the subjective nature of aesthetic assessments, even among expert neurosurgeons and craniofacial plastic surgeons who specialize in craniosynostosis. Objective measures to accurately assess phenotypical severity and aesthetic outcome after surgery are needed to compare the aesthetic outcome of different surgical techniques. Although the use of 3D photogrammetry has been increasing, we opted for using 2D photographs in the current study as standard 2D photographs can be easily obtained by any health care provider. In contrast, 3D photogrammetry can be difficult to access for clinicians as the use of 3D photogrammetry requires expensive equipment, expertise, and time investments for analysis.^16^ In addition, there is no consensus on how 3D photogrammetry should be analyzed. Future studies should investigate the use of 3D photogrammetry, which allows for objective shape analyses in relation to the current 2D photo score. By combining results from 3D photogrammetry shape analysis and the 2D photo score, the photo score items can be refined further and used alongside 3D photogrammetry. Previous studies have already shown that trained deep learning algorithms based on 3D stereophotographs can distinguish healthy subjects from patients with scaphocephaly accurately.^17^ Using large data sets of both scaphocephaly patients and healthy controls, cranial shape analyses can be developed to detect more subtle morphologic differences. This is essential as existing crude outcome measures such as cephalic index and head circumference will likely not discriminate between subtle morphologic differences after different surgical techniques. In addition, given the heterogeneous phenotypes of sagittal synostosis, detecting subtle differences preoperatively and postoperatively is helpful to determine the optimal treatment strategy for aesthetic outcome, which may require phenotype-specific treatment strategies. Studies on optimizing aesthetic outcome should be part of a larger body of work that focuses on functional outcomes such as neurocognitive development, behavioral outcomes, visual outcomes, and the development of intracranial hypertension, and the quality of life of both patients and their parents by investigating the impact of diagnosis, treatment, and follow-up.

## Conclusion

This is the first large-scale multicenter study in which expert craniofacial plastic surgeons and neurosurgeons investigated the use of a suture-specific photo score to assess the severity of phenotypical characteristics of sagittal synostosis. Agreement on phenotypical severity was suboptimal (fair to moderate) for individual photo score items. Overall, the agreement on the overall phenotype determined by the summed score of individual photo score items was the highest (substantial agreement). This study highlights the need for objective measures to assess the severity of the scaphocephaly phenotype to allow for future benchmarking of aesthetic outcome between different centers with different treatment protocols.

## Supplementary Material

SUPPLEMENTARY MATERIAL
