# Supplementary material for: A Photo Score For Aesthetic Outcome In Sagittal Synostosis: An ERN CRANIO Collaboration
Source: J Craniofac Surg. 2023 Sep 13;34(8):2279–83. doi: 10.1097/SCS.0000000000009732 (PMC10597420; doi:10.1097/SCS.0000000000009732)
Supplement: SUPPLEMENTARY MATERIAL [file scs-34-2279-s001.docx]

| **Sagittal synostosis photo score** | **View** |
| --- | --- |
| **Frontal bossing**  Pronounced and prominent forehead with abnormal bulging of forehead | Lateral |
| **Occipital bullet**  Abnormal bulging / pronounced and prominent occiput | Lateral |
| **Vertex line depression**  Posterior part of the skull; lower vertex indicates a more severe phenotype | Lateral |
| **Elongated appearance of the skull**  Abnormally long skull shape; A longer skull indicates a more severe phenotype. | Lateral  Bird’s eye |
| **Biparietal narrowness**  Abnormally narrow / tapered biparietal appearance;  More narrow biparietal appearance indicates a more severe phenotype | Anterior-posterior  Bird’s eye |
| **Temporal hollowing / pinching**  Abnormal temporal hollowing/pinching above the supraorbital bar on the lateral sides of the forehead | Anterior-posterior |
| **Overall phenotype**  Severity of the overall phenotype | All |

| **Scale** | **Normal** | **Mild** | **Moderate** | **Severe** |
| --- | --- | --- | --- | --- |

**Supplemental Table1. The sagittal synostosis photo score.** Each participant received the same instructions and was shown example photos. Particpants could use all views to assess each individual item, but were instructed in which view the item was best visible. Each individual item was scored according to the same four-point scale.

**Supplemental Table2. Interrater reliability**

| **Photo score item** | **Modified Fleiss ‘ Kappa statistic** | **95% bootstrap CI** | **Agreement strength ^*^** |
| --- | --- | --- | --- |
| Frontal bossing | 0.56 | 0.47, 0.64 | Moderate |
| Occipital bullet | 0.50 | 0.43, 0.57 | Moderate |
| Vertex line depression | 0.40 | 0.33, 0.48 | Fair |
| Elongated skull | 0.50 | 0.44, 0.56 | Moderate |
| Biparietal narrowness | 0.38 | 0.31, 0.45 | Fair |
| Temporal hollowing | 0.41 | 0.36, 0.47 | Moderate |
| Overall phenotype | 0.53 | 0.48, 0.59 | Moderate |

**Supplemental Table2A.** Interrater reliability of each item in the photo score. **^*^**Agreement strength on Landis and Koch scale.

| **Intraclass correlation coefficient type** | **ICC** | **95% CI** | **Agreement strength ^*^** |
| --- | --- | --- | --- |
| Agreement | 0.69 | 0.57, 0.82 | Substantial |
| Consistency | 0.78 | 0.67, 0.87 | Substantial |

**Supplemental Table2B.** Intraclass correlation coefficient for sum scores (excluding *‘Overall phenotype’*). **^*^**Agreement strength on Landis and Koch scale.

Abbreviations: ICC = intraclass correlation coefficient, CI= confidence interval.

**Supplemental Table3.** Spearman rank correlation between sub-items and overall phenotype.

Abbreviations: r_s_ = Spearman's rank correlation coefficient, CI = confidence interval.

| **Frontal bossing** | | |  | **Temporal hollowing** | | |
| --- | --- | --- | --- | --- | --- | --- |
| **Rater** | **r_s_** | **95% bootstrap CI** |  | **Rater** | **r_s_** | **95% bootstrap CI** |
| Rater 1 | 0.44 | 0.00, 0.73 |  | Rater 1 | 0.52 | 0.14, 0.75 |
| Rater 2 | 0.64 | 0.30, 0.82 |  | Rater 2 | 0.27 | -0.24, 0.62 |
| Rater 3 | 0.72 | 0.24, 0.92 |  | Rater 3 | 0.74 | 0.35, 0.90 |
| Rater 4 | 0.72 | 0.43, 0.87 |  | Rater 4 | 0.46 | 0.09, 0.69 |
| Rater 5 | 0.53 | 0.19, 0.73 |  | Rater 5 | 0.00 | -0.37, 0.38 |
| Rater 6 | 0.61 | 0.23, 0.80 |  | Rater 6 | 0.10 | -0.34, 0.47 |
| Rater 7 | 0.66 | 0.37, 0.84 |  | Rater 7 | 0.17 | -0.26, 0.59 |
| Rater 8 | 0.84 | 0.65, 0.94 |  | Rater 8 | 0.59 | 0.17, 0.82 |
| Rater 9 | 0.63 | 0.29, 0.81 |  | Rater 9 | 0.71 | 0.37, 0.86 |
| Rater 10 | 0.65 | 0.24, 0.87 |  | Rater 10 | 0.59 | 0.26, 0.79 |
| Rater 11 | 0.40 | -0.06, 0.71 |  | Rater 11 | 0.50 | 0.06, 0.74 |
| Rater 12 | 0.59 | 0.09, 0.79 |  | Rater 12 | 0.44 | 0.08, 0.68 |
| Rater 13 | 0.68 | 0.36, 0.87 |  | Rater 13 | 0.36 | -0.23, 0.69 |
| Rater 14 | 0.41 | -0.05, 0.69 |  | Rater 14 | 0.40 | -0.1, 0.69 |
| Rater 15 | 0.39 | 0.01, 0.65 |  | Rater 15 | 0.38 | 0.00, 0.68 |
| Rater 16 | 0.42 | 0.02, 0.73 |  | Rater 16 | 0.11 | -0.34, 0.56 |
| Rater 17 | 0.69 | 0.33, 0.87 |  | Rater 17 | 0.38 | -0.06, 0.64 |
| Rater 18 | 0.58 | 0.26, 0.79 |  | Rater 18 | 0.60 | 0.25, 0.78 |
| Rater 19 | 0.63 | 0.32, 0.81 |  | Rater 19 | 0.51 | 0.06, 0.78 |
| Rater 20 | 0.82 | 0.65, 0.92 |  | Rater 20 | 0.54 | 0.12, 0.77 |
| Rater 21 | 0.69 | 0.40, 0.84 |  | Rater 21 | 0.35 | 0.08, 0.68 |
| Rater 22 | 0.55 | 0.22, 0.77 |  | Rater 22 | 0.09 | -0.32, 0.53 |
| Rater 23 | 0.60 | 0.18, 0.84 |  | Rater 23 | 0.33 | -0.08, 0.62 |
| Rater 24 | 0.64 | 0.22, 0.83 |  | Rater 24 | 0.40 | 0.03, 0.66 |
| Rater 25 | 0.54 | 0.10, 0.78 |  | Rater 25 | 0.64 | 0.3, 0.82 |
| Rater 26 | 0.70 | 0.24, 0.89 |  | Rater 26 | 0.74 | 0.41, 0.88 |
|  |  |  |  |  |  |  |
| **Occipital bullet** | | |  | **Vertex line depression** | | |
| **Rater** | **r_s_** | **95% bootstrap CI** |  | **Rater** | **r_s_** | **95% bootstrap CI** |
| Rater 1 | 0.76 | 0.52, 0.91 |  | Rater 1 | 0.30 | -0.17, 0.64 |
| Rater 2 | 0.76 | 0.5, 0.88 |  | Rater 2 | 0.75 | 0.36, 0.89 |
| Rater 3 | 0.32 | -0.16, 0.70 |  | Rater 3 | 0.34 | -0.15, 0.62 |
| Rater 4 | 0.73 | 0.4, 0.87 |  | Rater 4 | 0.65 | 0.33, 0.82 |
| Rater 5 | 0.67 | 0.26, 0.84 |  | Rater 5 | 0.55 | 0.10, 0.83 |
| Rater 6 | 0.67 | 0.27, 0.86 |  | Rater 6 | 0.28 | -0.13, 0.60 |
| Rater 7 | 0.66 | 0.34, 0.84 |  | Rater 7 | 0.40 | 0.25, 0.64 |
| Rater 8 | 0.68 | 0.29, 0.87 |  | Rater 8 | 0.33 | -0.09, 0.61 |
| Rater 9 | 0.83 | 0.61, 0.91 |  | Rater 9 | 0.83 | 0.63, 0.93 |
| Rater 10 | 0.84 | 0.55, 0.92 |  | Rater 10 | 0.56 | 0.16, 0.79 |
| Rater 11 | 0.80 | 0.51, 0.90 |  | Rater 11 | 0.89 | 0.76, 0.95 |
| Rater 12 | 0.68 | 0.32, 0.86 |  | Rater 12 | 0.48 | 0.03, 0.73 |
| Rater 13 | 0.77 | 0.54, 0.89 |  | Rater 13 | 0.60 | 0.11, 0.86 |
| Rater 14 | 0.86 | 0.69, 0.93 |  | Rater 14 | 0.79 | 0.53, 0.90 |
| Rater 15 | 0.72 | 0.35, 0.88 |  | Rater 15 | 0.69 | 0.41, 0.85 |
| Rater 16 | 0.85 | 0.66, 0.95 |  | Rater 16 | 0.38 | 0.00, 0.65 |
| Rater 17 | 0.75 | 0.50, 0.88 |  | Rater 17 | 0.63 | 0.29, 0.83 |
| Rater 18 | 0.60 | 0.17, 0.80 |  | Rater 18 | 0.58 | 0.17, 0.80 |
| Rater 19 | 0.49 | 0.02, 0.79 |  | Rater 19 | 0.55 | 0.17, 0.75 |
| Rater 20 | 0.79 | 0.47, 0.92 |  | Rater 20 | 0.76 | 0.52, 0.88 |
| Rater 21 | 0.57 | 0.09, 0.83 |  | Rater 21 | 0.34 | -0.08, 0.61 |
| Rater 22 | 0.86 | 0.73, 0.95 |  | Rater 22 | 0.52 | 0.09, 0.77 |
| Rater 23 | 0.70 | 0.26, 0.86 |  | Rater 23 | 0.60 | 0.25, 0.82 |
| Rater 24 | 0.81 | 0.60, 0.89 |  | Rater 24 | 0.35 | -0.08, 0.69 |
| Rater 25 | 0.80 | 0.65, 0.89 |  | Rater 25 | 0.58 | 0.28, 0.79 |
| Rater 26 | 0.60 | 0.16, 0.80 |  | Rater 26 | 0.68 | 0.34, 0.87 |
|  |  |  |  |  |  |  |
| **Elongated skull appearance** | | |  | **Biparietal narrowness** | | |
| **Rater** | **r_s_** | **95% bootstrap CI** |  | **Rater** | **r_s_** | **95% bootstrap CI** |
| Rater 1 | 0.65 | 0.24, 0.88 |  | Rater 1 | 0.71 | 0.35, 0.88 |
| Rater 2 | 0.72 | 0.4, 0.90 |  | Rater 2 | 0.92 | 0.58, 1.00 |
| Rater 3 | 0.65 | 0.32, 0.84 |  | Rater 3 | 0.61 | 0.21, 0.82 |
| Rater 4 | 0.73 | 0.31, 0.89 |  | Rater 4 | 0.76 | 0.37, 0.90 |
| Rater 5 | 0.85 | 0.63, 0.91 |  | Rater 5 | 0.60 | 0.35, 0.80 |
| Rater 6 | 0.82 | 0.50, 0.92 |  | Rater 6 | 0.84 | 0.69, 0.93 |
| Rater 7 | 0.85 | 0.64, 0.94 |  | Rater 7 | 0.55 | 0.13, 0.78 |
| Rater 8 | 0.84 | 0.63, 0.93 |  | Rater 8 | 0.67 | 0.28, 0.84 |
| Rater 9 | 0.77 | 0.51, 0.88 |  | Rater 9 | 0.85 | 0.50, 0.97 |
| Rater 10 | 0.91 | 0.67, 0.98 |  | Rater 10 | 0.88 | 0.64, 0.95 |
| Rater 11 | 0.80 | 0.41, 0.91 |  | Rater 11 | 0.88 | 0.67, 0.94 |
| Rater 12 | 0.73 | 0.46, 0.88 |  | Rater 12 | 0.54 | 0.01, 0.79 |
| Rater 13 | 0.92 | 0.81, 0.98 |  | Rater 13 | 0.79 | 0.40, 0.92 |
| Rater 14 | 0.78 | 0.46, 0.88 |  | Rater 14 | 0.90 | 0.75, 0.97 |
| Rater 15 | 0.82 | 0.56, 0.91 |  | Rater 15 | 0.85 | 0.73, 0.92 |
| Rater 16 | 0.87 | 0.71, 0.97 |  | Rater 16 | 0.82 | 0.52, 0.94 |
| Rater 17 | 0.91 | 0.81, 0.97 |  | Rater 17 | 0.80 | 0.53, 0.92 |
| Rater 18 | 0.72 | 0.39, 0.86 |  | Rater 18 | 0.82 | 0.66, 0.92 |
| Rater 19 | 0.72 | 0.35, 0.89 |  | Rater 19 | 0.53 | 0.21, 0.72 |
| Rater 20 | 0.82 | 0.60, 0.91 |  | Rater 20 | 0.83 | 0.64, 0.91 |
| Rater 21 | 0.70 | 0.08, 0.90 |  | Rater 21 | 0.48 | -0.02, 0.81 |
| Rater 22 | 0.94 | 0.79, 0.99 |  | Rater 22 | 0.77 | 0.26, 0.92 |
| Rater 23 | 0.57 | 0.05, 0.83 |  | Rater 23 | 0.74 | 0.34, 0.90 |
| Rater 24 | 0.87 | 0.75, 0.94 |  | Rater 24 | 0.81 | 0.52, 0.91 |
| Rater 25 | 1.00 | 0.95, 1.00 |  | Rater 25 | 0.72 | 0.45, 0.88 |
| Rater 26 | 0.85 | 0.68, 0.95 |  | Rater 26 | 0.83 | 0.55, 0.93 |

**Supplemental Table4. Interrater reliability for high quality photosets**

| **Photo score item** | **Modified Fleiss ‘ Kappa statistic** | **95% bootstrap CI** | **Agreement strength** |
| --- | --- | --- | --- |
| Frontal bossing | 0.39 | 0.31, 0.46 | Fair |
| Occipital bullet | 0.55 | 0.44, 0.66 | Moderate |
| Vertex line depression | 0.34 | 0.26, 0.42 | Fair |
| Elongated skull | 0.50 | 0.41, 0.59 | Moderate |
| Biparietal narrowness | 0.32 | 0.25, 0.39 | Fair |
| Temporal hollowing | 0.39 | 0.31, 0.48 | Fair |
| Overall phenotype | 0.56 | 0.47, 0.66 | Moderate |

**Supplemental Table4A.** Interrater reliability of each item in the photo score for 10 optimal quality photosets. **^*^**Agreement strength on Landis and Koch scale.

| **Intraclass correlation coefficient** | **ICC** | **95% CI** | **Agreement strength** |
| --- | --- | --- | --- |
| Agreement | 0.56 | 0.36, 082 | Moderate |
| Consistency | 0.67 | 0.48, 0.87 | Moderate |

**Supplemental Table4B.** Intraclass correlation coefficient for sum scores (excluding *‘Overall phenotype’* ) 10 optimal quality photosets. **^*^**Agreement strength on Landis and Koch scale.

Abbreviations: ICC = intraclass correlation coefficient, CI= confidence interval.
